# Bond Strength of Orthodontic Brackets to Temporary Crowns: In Vitro Effects of Surface Treatment

**DOI:** 10.1155/2021/9999933

**Published:** 2021-06-29

**Authors:** Suliman Y Shahin, Tahani H. Abu Showmi, Sadeem H. Alzaghran, Hoda Albaqawi, Latifah Alrashoudi, Mohammed M. Gad

**Affiliations:** ^1^Department of Preventive Dental Sciences, College of Dentistry, Imam Abdulrahman Bin Faisal University, P. O. Box 1982, Dammam 31441, Saudi Arabia; ^2^College of Dentistry, Imam Abdulrahman Bin Faisal University, P. O. Box 1982, Dammam 31441, Saudi Arabia; ^3^Department of Substitutive Dental Sciences, College of Dentistry, Imam Abdulrahman Bin Faisal University, P. O. Box 1982, Dammam 31441, Saudi Arabia

## Abstract

**Background:**

The number of patients seeking orthodontic treatment has been consistently increasing. During orthodontic treatment, it is recommended to place the provisional restoration and to delay the final restoration until completion of orthodontic treatment. Recurrent bracket debonding necessitates orthodontists to prepare the bonding area with special measures.

**Objective:**

This study aimed to evaluate the effect of different grit sizes of diamond burs and sandblasting surface treatment on the shear bond strength of orthodontic brackets to provisional crowns.

**Materials and Methods:**

A total of 75 discs were fabricated from a bisacrylic composite and divided into 5 groups (*n* = 15) according to surface treatment by black, blue, and green diamond burs and sandblasting in addition to a control group. Metal orthodontic brackets were bonded to discs in a standardized conventional manner. All specimens were subjected to thermocycling with 5000 cycles of alternating 5°C and 55°C waterbaths. The shear bond strength test was performed using a universal testing machine. A scanning electron microscope (SEM) was used to analyze the surface treatment effect and features of debonded surfaces. The amount of composite resin left on the specimen surfaces was analyzed and classified with the adhesive remnant index. One-way ANOVA was performed at *α* = 0.05.

**Results:**

The shear bond strength of specimens treated with sandblasting was significantly higher than that of the control group under thermal aging conditions (*p*=0.022), as well as blue burs (*p*=0.001), while no significant differences were found between different grit diamond burs and the controls (*p* > 0.05).

**Conclusion:**

Under thermocycling conditions, sandblasting of provisional crowns increases the bond strength of orthodontic brackets.

## 1. Introduction

Orthodontic bonding of brackets to teeth is a standard procedure to align teeth with fixed appliances [1]. A substantial number of patients seek orthodontic treatment for various reasons, which in turn has caused many clinical challenges during orthodontic therapy [2]. For example, in many cases, orthodontic treatment is delivered in conjunction with other specialties to facilitate restorative therapy of other dental professions, such as periodontics and prosthodontics [3, 4]_._ Furthermore, it is common to encounter a patient with provisional crowns who seeks orthodontic treatment to alleviate anterior spacing resulting from missing teeth [5].

In prerestorative orthodontic treatment, clinicians bond orthodontic brackets to natural teeth as well as teeth that have extensive composite resin restorations and ceramic or provisional resin crowns [6]. In cases of tooth fracture or gross carious lesions extending subgingivally, a provisional crown is needed prior to orthodontic root extrusion to avoid biological width violation in the permanent crown [7]. A provisional crown serves to provide temporary protection, stabilization, and function of the tooth, as it also determines the esthetic outcome of the final restoration [7, 8]. In such a clinical situation, an orthodontic bracket is bonded to the provisional crown material (PCM) [7].

Many materials are available on the market for provisional crown fabrication, such as polymethylemethacrylate (PMMA) resin and bisacryl composite resin. The best recommended PCM is bisacryl composite resin, as it showed low exothermic reaction during setting, better strength, color stability, and adequate marginal adaptation when compared to PMMA [9]. Orthodontic tooth movement requires different amounts of force to achieve clinical orthodontic movement. The ranges of force needed for translatory and extrusive movements are 70–120 g and 35–60 g, respectively. According to Reynolds, the minimum suggested tensile bond strength between orthodontic brackets and teeth is 6–8 MPa, which is required for clinical orthodontic tooth movement [10].

Orthodontists are concerned about the bond strength between bracket adhesive and synthetic surfaces in clinical practice [11]. A weak bond of the brackets to provisional materials will lead to a high failure rate, with adverse consequences on the cost and efficiency of orthodontic therapy and on patient comfort [3, 11]. This concern has been addressed by several methods, including surface treatment by mechanical or chemical approaches to roughen the surface and increase the surface area for bonding. Mechanical preparation includes sandblasting and surface grinding with carbide burs or diamond burs. Chemical methods include etching with phosphoric acid and hydrofluoric acid [12].

A study conducted in 2007 by Siew et al. showed that the shear bond strength was influenced by surface treatment and the duration of aging [9]. In groups bonded after one week of specimen fabrication, the surface-treated groups have significantly higher bond strength than the control group. In groups bonded after one month, compared to the control group, the surface-treated groups showed lower shear bond strength. However, the group treated with green stone had significantly higher shear bond strength than the sandblasting group when bonded to the orthodontic bracket within one week [9]. Although the bond strength of orthodontic brackets to temporary crown material has been investigated in previous studies using different surface treatments, in comparison to conventional treatment, the effect of different grit sizes of diamond bur treatment has not been investigated before. Therefore, the current study aimed to evaluate the shear bond strength between orthodontic brackets and provisional crown material using different mechanical surface treatments.

## 2. Materials and Methods

Power analysis for this study using a significance level of *α* = 0.05 and power = 0.80 was calculated using the study by Rambhia et al. [7]. The difference between the two means for the success CD group using Fuji Ortho LC brackets was chosen as the biggest difference in mean share bond strength in MPa (9.33, 7.42) SD = 1.73. Minimum sample size per group needed is 13. A sample size of 15 per group was found to be sufficient.

A total of 75 cylindrical specimens (diameter, 10 mm; height, 15 mm) of bisacrylic composite material (cold curing temporary crown material; success CD, Neumünster/Germany) were prepared according to the manufacturer's instructions. Bisacrylic material supplied in automix cartridges injected in a standardized silicon mold (10 mm^*∗*^15 mm) was then pressed with a glass slab to extrude the excess material. Set by using light cure (Acteon Satelec mini LED light cure) at rapid mode intensity (1250 mW/cm^2^) with the end of the optical guide of the light cure positioned directly to the specimen for 30 seconds for each sample. Afterward, all the specimens were stored at room temperature in distilled water at 3°C for 24 hours. The 75 specimens were randomly divided into 5 groups (*n* = 15) according to surface treatment (Table 1).

Stainless steel mandibular central incisor brackets (Gemini Metal Brackets; 3 M Unitek) were bonded to the provisional material specimens with a light-cured composite resin (Transbond XT; 3 M Unitek) by the same investigator. The provisional material specimens were etched with 37% phosphoric acid for 30 seconds, rinsed with water spray for 20 seconds, and dried with compressed air for another 20 seconds. Stainless steel mandibular incisor brackets were bonded to the provisional material specimens with a light-cured composite resin in the base of the brackets according to the manufacturer's instructions. Any excess adhesive will be removed with the aid of a probe, and the adhesive will be light-cured by means of an Ortholux XT visible light-curing unit (3 M Unitek) for 10 seconds on the mesial and distal sides (5 seconds on each side), according to the manufacturer's instructions. Once bracket bonding had been finished, the specimens will be stored in distilled water at 37°C for 24 hours. All specimens were subjected to a thermocycling procedure applying 5000 cycles of alternating 5°C and 55°C waterbaths. The specimens were incubated for 30 seconds in cold or hot water with a 5-second interval between successive immersions, employing a thermocycling machine (Thermocycler THE-1100-SD Mechatronik GmbH, Feldkirchen - Westerham, Germany).

**Figure 1:**
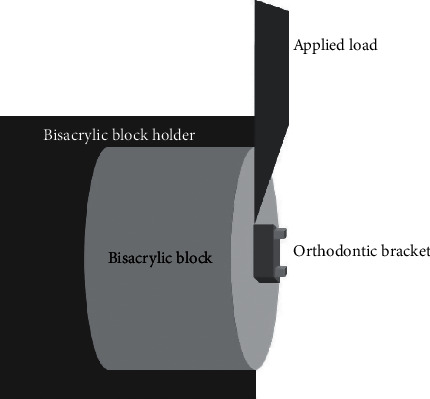
Illustrated diagram for the shear bond strength test.

The specimens were mounted on the specimen fixture on the universal testing machine (Instron 8871; Instron Co., Norwood, MA), as illustrated in Figure 1. A knife-edge blade at the bracket-specimen interface was loaded at a speed of 1.0 mm/min until bond failure occurred. The load at failure was converted into mega-Pascal units (MPa) according to the following equation: shear bond strength = *F*/A (N/mm^2^ or MPa), where *F* is the debonding force in Newton, and *A* is the cross-sectional surface area of the bracket base in square millimeters [12].

The effect of surface treatment and features of debonding surfaces were analyzed using SEM (FEI, Inspect S50, Czech Republic). The specimens were sputter coated (Quorum, Q150 R ES) and scanned at 20 kV with a working distance of ∼10 mm. Representative SEM images were recorded at different magnifications to examine the effect of surface treatment and to investigate homogeneity/porosity at the fractured surfaces and the mode of failure.

To evaluate the bond failure interface, the debonded provisional material and bracket bases were inspected visually and then under an optical microscope (Nikon, H550 L, Tokyo, Japan) at 10-fold magnification. After debonding, the nature of failure was classified according to the adhesive remnant index (ARI) score regarding the residual adhesive on the bonded area of the provisional materials as follows: 0 = no adhesive left on the crown, 1 = less than 50% of adhesive left on the crown, 2 = more than 50% of adhesive left on the crown, and 3 = 100% of adhesive left on the crown with a distinct impression of the bracket mesh [13, 14].

Data analysis was performed by using SPSS-20.0 (IBM product, Chicago, USA). Numerical data based on measurements of shear bond strength into groups, consisting of control, blue, green, black, and sandblasting, were presented as the mean (standard deviation) and tested for normality within each group using the Kolmogorov–Smirnov test, and the sample was found to be normally distributed. One-way ANOVA was performed to compare the results of shear bond strength between the groups. A *p* value ≤0.05 was considered significant.

## 3. Results

The mean, standard deviation (SD), and significance of shear bond strength are summarized in Table 2. The mean shear bond strength measured in the control group was recorded at 9.79 ± 4.96 MPa, which was comparatively higher but nonsignificant with the mean recorded in the blue bur, 7.13 ± 3.31 MPa. However, it was less than the mean recorded in the green bur, black bur, and sandblasting 11.48 ± 7.40 MPa, 12.42 ± 7.59 MPa, and 17.78 ± 9.99 MPa, respectively. One-way ANOVA revealed a mean significant difference among the five groups (*F* = 4.70, *p*=0.002), but a significant difference in shear bond strength was seen only with regard to sandblasting in the comparison of the control (*p*=0.022) and blue (*p*=0.001) groups as illustrated in Table 2.

**Figure 2:**
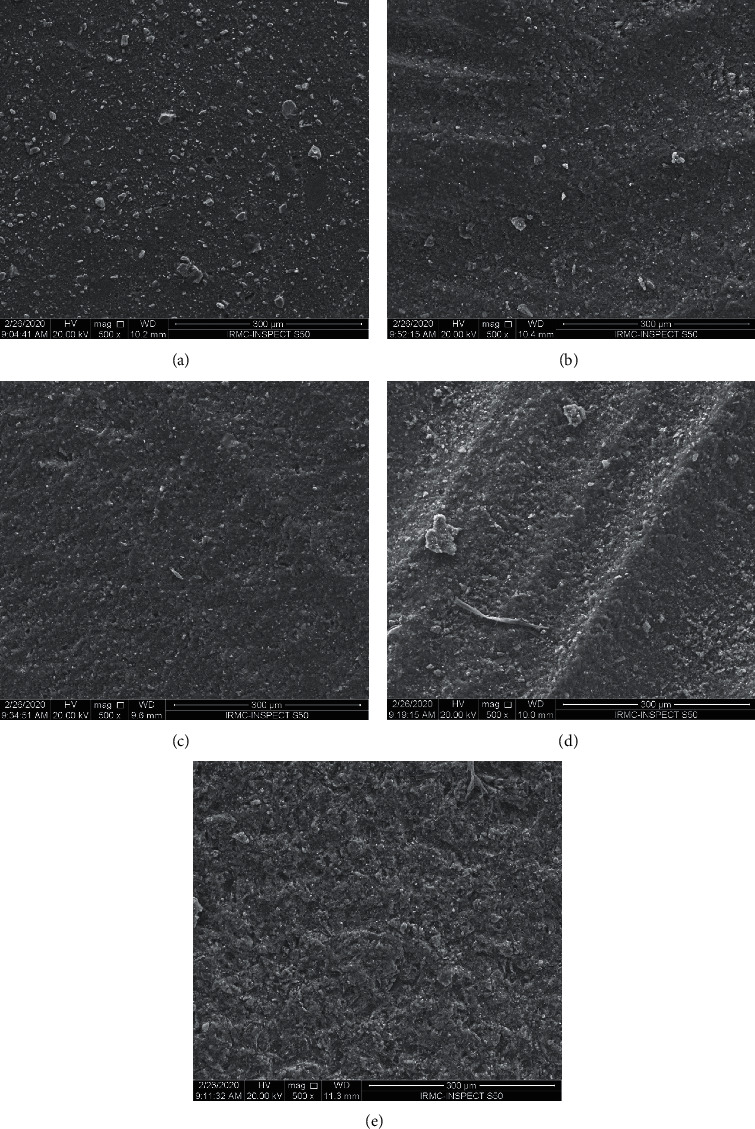
(a), (b), (c), (d), (e) SEM representative images for surface treatment effects.

Figure 2 shows representative SEM images of the surface treatment effect. A smooth surface was displayed in the control group (Figure 2(a)). The smooth to rougher surfaces are shown in Figures 2(b)–2(e), where bur treatment showed some irregularities corresponding to bur flutes, which are clearly displayed with black burs (Figure 2(d)). The surface topography changed with sandblasting treatment, where homogenous irregularities were displayed with sandblasting (Figure 2(e)).

The nature of failure of deboned specimens is summarized in Table 3. For the control group, the most common fracture type was adhesive fracture (ARI score 0) between provisional material and adhesive. The nature of failure of the blue bur group was close to that of the control group (ARI score 0). For the other three groups (green bure, black bur, and sandblasting), a dramatic change in the nature of failure displayed the most debonded surfaces with an ARI score of 3. Although adhesive failure was the dominant fracture type with different surface treatments, the dominant adhesive failure was at the metal interface.

**Figure 3:**
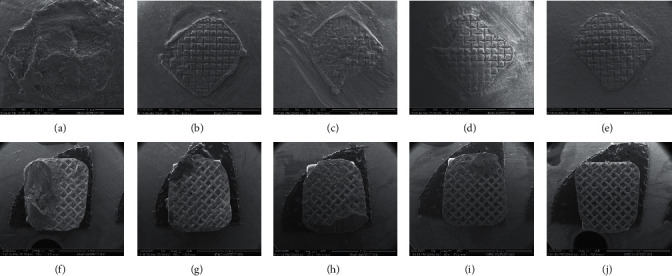
Representative SEM images for deboned surface after the SBS test. (a–e) Crown surfaces; (f–j) bracket surfaces. (a) Control, (b) blue bur, (c) green bur, (d) black bur, and (e) sandblasting bur.

Representative SEM images of the specimen surfaces after the SBS test are shown in Figure 3 (3(a)–3(e) show the crown side, while 3(f)–3(j) show the metal side). In the control group, complete separation of the adhesive illustrated a bracket surface where the mesh side was filled with adhesive (Figure 3(f)) and completely detached from the crown surface (Figure 3(a)). SEM analysis specimens with surface treatment showed different behavior where the bonding failure occurs partially between the metal and the adhesive (Figures 3(b), 3(c) and 3(f), 3(g)), while in these samples, it shifted to complete failure at the metal-adhesive interface (Figures 3(d), 3(e) and 3(i), 3(j)).

## 4. Discussion

Adequate retention of orthodontic fixed appliances throughout the full course of treatment plays a major role in the success of rendering orthodontics, rather than having a course of treatment that involves multiple bracket debonding incidences [13]. This study was conducted to evaluate and to compare the shear bond strength between orthodontic brackets and provisional crown material using common mechanical surface treatment protocols. The bisacrylic composite is recommended for provisional crowns over other provisional materials in cases that require combined prosthetic and orthodontic treatment for the long-term because of their superior mechanical properties and strength [3, 15]. Hence, this material was used in this research. The bond strength between orthodontic brackets and provisional crown material should be strong enough to tolerate orthodontic forces without debonding; however, at the end of the orthodontic treatment, the brackets should be easily removed [12]. The magnitude of bracket bond strength needed to withstand orthodontic forces throughout the treatment is hard to determine in various oral conditions.

It has been reported that the minimum required shear bond strength ranges between 6 and 8 MPa, which is important to maintain the bond of the orthodontic brackets to provisional crown material [3]. Some studies estimated a range of 6.5–10 MPa for SBS [16]. Based on the results of this study, the mean shear bond strength values of the surface-treated groups were within the range of clinically accepted bond strength. Furthermore, the different surface treatments affected the shear bond strength of orthodontic brackets to provisional crown material. Therefore, our null hypothesis was rejected.

The type of surface treatment technique of the provisional crown material was a crucial factor influencing the shear bond strength values in our specimen [12]. Diamond burs and sandblasting were chosen because they had been clinically used for surface roughening in the past [11]. Orthodontists commonly use diamond burs for surface preparation on provisional crowns before bracket bonding [17]. Dias FM et al. [17] investigated the effects of different surface pretreatments, including diamond burs, and reported that shear bond strength with diamond bur treatment exhibited higher values than the untreated group. Based on scanning electron microscopy findings of surface treatment, in comparison to the control group, the increase in shear bond strength values with diamond bur roughening could be attributed to the fact that the mechanical abrasive methods that increase mechanical interlocking are perhaps the most significant factor contributing to bond strength [3, 12]. This result can also be explained by the formation of deep craters and streaks that are appropriate for micromechanical retention of orthodontic adhesives. However, when compared to the control group, the increase in shear bond strength values with different grit sizes of diamond burs was not significant. On the other hand, to our knowledge, no study has investigated the surface modification by using different grit sizes of diamond burs on the shear bond strength of bisacrylic composites.

Surface roughening with sandblasting yields a significant increase in shear bond strength, which can be explained by an increase in the interface between the two surfaces that allows for a more bonded surface area. Another study has reported that sandblasting with alumina particles (microetcher) produces the highest bond strength among other surface treatments [18]. These values were also much higher than the minimum recommended 5–8 MPa, as reported by Reynolds. Furthermore, acrylic or composite resin maintains bond strength above the level accepted for clinical use [10, 19]. Two studies have found that a range of 4–10 MPa is the required SBS for orthodontic treatment [20, 21]. Since, higher forces applied to temporary crowns may not pose undesirable side effects such as natural teeth due to the absence of biological enamel structure. In our experiment, exceeding the recommended shear bond strength would not cause any undesirable effect to the provisional crown material.

Thermocycling intends to thermally stress the adhesive joint interface [13]. Al Jabbari et al. [3] reported that the differences in the coefficient of thermal expansion between the adhesive and the bisacrylic composite lead to changes in the volume of the materials during temperature fluctuations, which end with microleakage and fatigue of the joints between the two materials. Sandblasting exhibited an increase in shear bond strength, which may be attributed to the presence of bifunctional acrylates that cross-link to produce higher mechanical strength and decrease weakening in the presence of water [14, 22]. This outcome is in agreement with Al Jabbari et al. [3] who showed the same unexpected result of increased shear bond strength of bisacrylic composite (Protemp) in sandblasted groups after thermal aging. This finding could be explained by the increased mobility of radicals, which would provide additional polymerization during heating in the thermal aging procedure [3]. Our results were similar to those of Al Jabbari et al. [3], in addition to the findings reported by Blakey et al. [2] and Almeida et al. [14]. The nature of the failure in the present study was visually evaluated followed by light microscope examination. For the control group and blue group (ARI score 0), adhesive failure was the dominant type, demonstrating that no or less than half of adhesive persistent on the provisional materials where bonding failures occurred between adhesive and provisional crown material, indicating weak bond strength. However, when comparing the green bur, black bur, and sandblasting surface treatments, we found that all of the adhesive failures shifted between the adhesive and the bracket, which indicates an improvement in the bond strength between the adhesive and provisional crown (ARI score 3). In agreement with a previous study [13] investigating the effect of surface sandblasting, we found that most of the failures in the metal orthodontic bracket group were classified as cohesive, where all or more than half of the cement sustained on the provisional crown.

Based on our findings, sandblasting of the bisacrylic temporary crown would be a recommended surface treatment for increased bond strength, since it may improve the bonding strength of orthodontic brackets to the crowns and minimize recurrent debonding of metal orthodontic brackets during orthodontic treatment. Sandblasting can be readily used chairside before bonding of orthodontic brackets. Clinicians are advised to sandblast the bracket area before cementing the provisional crown to ensure a good bonding bracket throughout orthodontic treatment.

Although wet conditions that mimic the oral environment and different mechanical surface treatments were used in this study, only one commercial provisional crown material was used. In addition to material limitations, only shear stress was simulated. Moreover, the specimen shape did not represent the actual provisional crown form. Therefore, further studies under conditions that imitate the oral environment with different provisional materials and different surface treatments are recommended for future studies.

## 5. Conclusions

Within the limitations of our study, the following conclusions were drawn:

  Under thermocycling conditions, sandblasted provisional crowns would increase the bond strength of orthodontic brackets
  Under thermocycling conditions, surface grinding of provisional crowns by supercoarse diamond burs could increase the bond strength of brackets to tolerate limited orthodontic forces

## Figures and Tables

**Table 1:** Specimens grouping according surface treatment and treatment procedures.

Group (*n*)	Surface treatment	
Control (15)	No treatment	
G1 (15)	Blue, medium grit grinding	Each specimen was subjected to 5 strokes in the same direction at the center of the surface by one well-trained investigator using diamond burs (Jota diamond bur, Switzerland) with three different grit sizes: medium, coarse, and supercoarse.
G2 (15)	Green, coarse grit grinding	
G3 (15)	Black, supercoarse grinding	
G4 (15)	Sandblasting	Specimens were sandblasted (50 mg alumina particles) for 10 seconds from a distance of 10 mm by the application of 0.55 MPa of propulsion pressure using Wassermann Dental-Machine, CEMAT-NT3, GMBH, Hamburg, Germany. Specimens were rinsed with a steady stream of water for 30 seconds and then dried with compressed air.

**Table 2:** One-way ANOVA for mean comparison of shear bond strength (MPa) among the groups.

Comparison	Groups					ANOVA
	Control (*n* = 15)	Blue (*n* = 15)	Green (*n* = 15)	Black (*n* = 15)	Sandblasting (*n* = 15)	
Mean (S. D)	9.79 (4.96)	7.13 (3.31)	11.48 (7.40)	12.42 (7.59)	17.78 (9.99)	*F* = 4.70
Vs/control	--	*p*=0.839	*p*=0.964	*p*=0.844	*p*=0.022^*∗*^	*p*=0.002^*∗*^
Vs/blue	--	--	*p*=0.445	*p*=0.251	*p*=0.001^*∗*^	
Vs/green	--	--	--	*p*=0.996	*p*=0.114	
Vs/black	--	--	--	--	*p*=0.237	

^*∗*^The mean difference is significant at the 0.05 level.

**Table 3:** Scores for the adhesive remnant index of all groups (*n* = 15) according to optical microscope (16x) analysis.

Groups	Score 0	Score 1	Score 2	Score 3
Control	8	2	4	1
Blue	7	3	4	1
Green	0	2	5	8
Black	0	4	2	9
Sandblasting	0	1	5	9

Score 0, no adhesive left on the crown surface. Score 1, less than half of the adhesive left. Score 2, more than half of the adhesive left. Score 3, all adhesive left on the crown surface, with a distinct impression of the bracket mesh.

## Data Availability

The data used to support the findings of this study are available from the corresponding author upon request.
